# Understanding treatment decision making in juvenile idiopathic arthritis: a qualitative assessment

**DOI:** 10.1186/1546-0096-11-34

**Published:** 2013-09-30

**Authors:** Ellen A Lipstein, William B Brinkman, Jessica Sage, Carole M Lannon, Esi Morgan DeWitt

**Affiliations:** 1 Department of Pediatrics, University of Cincinnati College of Medicine, Cincinnati, OH, USA; 2 Division of Adolescent Medicine, Cincinnati Children’s Hospital Medical Center, 3333 Burnet Ave, MLC 7027, 45229 Cincinnati, OH, USA; 3 James M. Anderson Center for Health Systems Excellence, Cincinnati Children’s Hospital Medical Center, Cincinnati, OH, USA; 4 Division of General and Community Pediatrics, Cincinnati Children’s Hospital Medical Center, Cincinnati, OH, USA; 5 Division of Rheumatology, Cincinnati Children’s Hospital Medical Center, Cincinnati, OH, USA

**Keywords:** Juvenile idiopathic arthritis, Decision making, Biologics

## Abstract

**Background:**

The increase in therapeutic options for juvenile idiopathic arthritis (JIA) has added complexity to treatment decisions. Shared decision making has the potential to help providers and families work together to choose the best possible option for each patient from the array of choices. As part of a needs assessment, prior to design and implementation of shared decision making interventions, we conducted a qualitative assessment of clinicians’ current approaches to treatment decision making in JIA.

**Methods:**

Pediatric rheumatology clinicians were recruited from 2 academic children’s hospitals affiliated with a quality improvement learning network, using purposive and snowball sampling. Semi-structured interviews elicited how clinicians with prescribing authority (n = 10) interact with families to make treatment decisions. Interviews were audio-recorded and transcribed verbatim. A multi-disciplinary research team used content analysis to analyze the interview data.

To validate data from individual interviews and enrich our understanding, we presented the interview results to pediatric rheumatology clinicians attending a learning network meeting (n = 24 from 12 children’s hospitals). We then asked the clinicians questions to further identify and discuss areas of variation in the decision-making processes.

**Results:**

Clinicians described a decision-making process in which they, rather than the family or other care team members, consistently initiated treatment decisions. Initial treatment options presented to families generally reflected the clinician’s preferred treatment approaches, which differed across clinicians. Clinicians used various methods to inform families about treatment options and tailor information according to perceptions of a family’s information needs, level of comprehension or mood (e.g. anxiety). The attributes of medication presented to families fell into 4 categories: benefits, risks, logistics and family preferences. Clinicians typically included family members in the decision to initiate JIA treatment after limiting the options to fit the clinical situation and the clinician’s own preferences. Family members’ preferences were seen as more integral in the decision to stop treatment after symptom remission.

**Conclusions:**

Decision making about initial JIA treatment appears to be largely driven by clinician preferences. Family preferences are more likely to be considered for treatment discontinuation. Opportunities exist to develop, test, and implement tools to facilitate shared decision making in pediatric rheumatology.

## Background

Therapeutic options to treat juvenile idiopathic arthritis (JIA) have grown in number and complexity. What used to be a crippling condition is now a manageable though chronic one. Methotrexate has long been a foundational treatment for JIA, but there is substantial practice variation in its use [1-3]. In recent years, a growing number of other treatments have become available for JIA. Such treatments may differ in mechanism of action, dosing interval, mode of administration and safety profile [4]. To address this rapidly changing field, in 2011 the American College of Rheumatology published the first recommendations for the initiation of therapeutic agents for JIA [5]. The level of evidence behind these guidelines leaves multiple reasonable options, which differ in ways that matter to families [5,6]. Therefore, even with such guidelines, there is often a need to personalize treatment plans based upon unique factors brought to each interaction between a prescribing clinician and a patient.

In the face of uncertainty about which treatment is optimal for a specific patient [7], the decision-making process may be influenced by clinician training and practice style, clinician beliefs about the tradeoffs of safety versus effectiveness, and the stated or assumed preferences of patients and parents [8]. In the adult setting, the priorities of rheumatoid arthritis patients and rheumatologists can differ when making decisions about care escalation [9]. In pediatrics, many parents of children being treated for JIA are left with persistent questions, information needs, long-lasting concerns and worry about treatment adverse effects [6], suggesting a need for improved clinician-parent communication. Moreover, adolescents with JIA who have participated in treatment decisions often wish their role in the decision process had been different [10]. Their desire to be involved in decision making [11,12], may further complicate the process if their preferences differ from those of their parents [13].

Shared decision making, in which clinicians and families work together to make treatment decisions, may help overcome some of these challenges. This process typically involves clinicians offering options and describing the risks and benefits, and the patient and/or family members expressing their preferences and values [14]. This conversation leads to a better understanding of the relevant factors and shared responsibility for deciding how to proceed in a manner consistent with family preferences and values [15].

In previous work we elicited parent and adolescent perspectives regarding the treatment decision making in JIA [6,10]. However, the clinician perspective was unknown. Therefore, we undertook a needs assessment aimed at delineating the current decision making process in JIA treatment, in order to understand the barriers and facilitators to shared decision making. The study occurred within the context of a quality improvement initiative of the Pediatric Rheumatology Care and Outcomes Improvement Network (PR-COIN) aimed at enhancing shared decision making around therapeutics for JIA. PR-COIN is a learning network with a mission to improve the care delivery and outcomes of patients with JIA [16,17]. PR-COIN’s approach is to use quality improvement science in tandem with the improving chronic illness care model [18]. Central to this model is an enriched partnership of patients and practice teams to improve patient centered outcomes through the safe use of therapeutics and delivery of high quality care.

## Methods

### Design

We conducted a decision-making needs assessment in 2 steps. First we conducted and analyzed semi-structured interviews using established qualitative methods. Then we validated that data and enriched our understanding by presenting pediatric rheumatology clinicians attending a PR-COIN learning network meeting with questions to identify and discuss areas of variation, between clinicians, in the decision-making process. The Cincinnati Children’s Hospital Medical Center Institutional Review Board approved this study.

### Interview sample

Using a combination of purposeful and snowball sampling [19], we recruited pediatric rheumatology clinicians with prescribing authority from 2 large, academically-oriented children’s hospitals affiliated with PR-COIN. Using snowball sampling [19], where individuals identify people known to them to approach for study participation, we asked early participants to name fellow clinicians with disparate approaches to working with families. Our overall goal was to hear about diverse approaches to working with families. We e-mailed potential participants to introduce the project and inform them that a study team member would be calling, unless they declined to participate. A study team member then called potential participants to schedule an in-person interview with one of two trained interviewers (EL, JS). Informed consent was obtained from all participants. Interviews were audio-recorded for verbatim transcription and verified by study staff. Recruitment continued until information saturation was achieved [19].

### Interview structure

A semi-structured interview guide (Additional file 1) was developed based on adult and pediatric literature about treatment decision making, research on parents’ experiences with treatment decision making in JIA [6] and input from pediatric rheumatologists. As interviews progressed, the major content areas remained constant but changes were made based on information learned in prior interviews. Although several interviewees mentioned other treatment-related decisions, such as physical therapy and sports participation, we focused this needs assessment on decisions about medications. Interviews focused on understanding the typical decision-making process, treatment attributes (e.g., side effects, cost, time to improvement, etc.) considered during decision making and the challenges of specific decision-making situations (e.g., new diagnosis, poorly controlled symptoms, etc.).

### Interview data coding and analysis

We used descriptive content analysis for data coding and analysis [20,21]. After reviewing the initial transcripts, the research team identified major content categories. Each interview was then coded by a minimum of 2 members of the research team. Coders identified interview content that fit into the major categories, as well as additional concepts that had not been identified in the initial interviews. Codes were aggregated and compared between coders. Major differences were resolved through discussion. The analytic process sought to identify areas of agreement among interviews, as well as outlying concepts and variations between participants.

### Data validation

Validation refers to the process of presenting the investigators’ findings to the participants or to individuals with similar characteristics to confirm the findings [20]. We first did this by giving a presentation of the findings from our interviews to the pediatric rheumatology professionals attending a PR-COIN collaborative learning session. A learning session is a face-to-face meeting of learning network teams to study quality improvement, share results, and advance network projects. To identify and discuss areas of variation in decision-making processes, particularly related to the attributes found to influence decision making during initial interviews, we constructed multiple choice questions based upon the interview data (Additional file 2) and used an audience response system to capture the pattern of responses. For example, we asked, “How often do you discuss the magnitude of the expected improvement when starting or changing mediations: never, rarely, sometimes, almost always, always?” Following each question, the percent choosing each option was displayed graphically to participants and research team members facilitated a discussion to better understand the sources of variation among participants. This process assisted in the organization of themes from the interviews and confirmed our results. Results were further validated by inviting review and feedback on a draft of this manuscript by both interview and learning session participants. Collectively these groups are referred to as clinicians throughout this manuscript.

## Results

### Participants

We interviewed a total of 10 clinicians. Most were white, female and had been in their current position for an average of 4.5 years (Table 1). Learning session participants included 24 clinicians from 12 hospitals, of which 21 had prescribing authority. Demographic information was not collected from learning session participants, some of whom had also participated in interviews.

**Table 1:** Interview participants

**Age**	
Median (Range)	52.5 years (31–66)
**Gender**	**n (%)**
Male	4 (40%)
Female	6 (60%)
**Race**	**n (%)**
White/Caucasian	8 (80%)
Asian	2 (20%)
**Role**	**n (%)**
Physicians	8 (80%)
Attending	6
Fellow	2
Certified Nurse Practitioner	2 (20%)
**Years in current position**	
Median (Range)	4.5 years (2–25)

Our results are organized according the four major themes found in our data: clinicians’ decision process, treatment attributes, working with families to make decisions, and challenges in JIA treatment decisions. Each section has a corresponding table with quotations illustrating the findings.

### Clinicians’ decision process

All clinicians indicated that the chronicity of JIA leads to multiple decision points. (Table 2) Moreover, decisions may recur during the course of treating a patient with JIA. As one clinician stated, “…down the road, we need to have a discussion and see how much benefit [the patient’s] gotten….then it’s just a reassessment.” In considering medication decisions most clinicians reported they had a preferred approach to treatment, such as following published guidelines, step-up therapy, or early aggressive therapy. However, there was wide-variation in which of these approaches each individual favored. These individual preferences, in turn, determined the menu of options presented to families.

**Table 2:** Clinicians’ decision process

**Sub-themes**	**Supporting quotations**
Preferred Treatment Approach	“I typically sort of set up a menu of possibilities, all of which are within that evidence-based realm, and I’m fairly comfortable with choices they make.” *Clinician 9*
	“Well, so in my own mind, I have a certain approach I use… it’s divided up into kind of immediate short-term treatment and then also treatments that would address long-term disease control.” *Clinician 10*
	“So it all depends on how they present, what phase they get to us, how many joints, how much disability, how much systemic disease, and then try to be aggressive at first to get the thing under control.” *Clinician 12*
	“…now there’s treatment guidelines, so that makes it easier. I mean, kind of a step-wise approach.” *Clinician 14*
Timing of Decision	“So after diagnosis has been reached I go through the different types of medications that we use to and I usually start by talking about the more benign medications.” *Clinician 2*
	“A treatment plan can always change … whether it’s side effects or flare-up of disease or something like that or new medical literature that comes out.” *Clinician 3*
	“If it’s recurrence of disease after a long period of being quiet, you need to know what the family has been through before…” *Clinician 9*

The clinician’s preferred approach sometimes depended on the timing of the decision: whether the decision occurs at diagnosis, when symptoms persist despite current treatment, or when a patient has clinical remission on treatment. When initiating treatment for newly diagnosed children and adolescents, clinicians felt the treatment discussion was typified by a presentation of the array of treatment options perceived by the clinician to be the best choices. In the setting of treatment failure, decision making was complicated by the need to distinguish between ineffective treatment, poor adherence and treatment-limiting adverse drug reactions. After achieving remission, “…the attention immediately turns to ‘How do we get off these things?’”

### Treatment attributes

When presenting treatment options to families, the attributes considered by clinicians fell into 4 categories: benefits, risks, logistics and family preferences. (Table 3) Treatment benefits included symptom relief, long-term effectiveness and time to expected improvement. As one physician stated, in describing a particular patient encounter, “I wanted to start [a specific medication] because I knew that it would work a lot faster.” Nearly all clinicians said they always or almost always discussed how likely a medication was to work, but there was less consistency in discussing the expected magnitude of improvement. In contrast, all clinicians considered the risks of treatment and discussed side-effects with parents. Infusion reactions, immunosuppression and malignancy were specifically mentioned as important side-effects to discuss.

**Table 3:** Treatment attributes

**Sub-themes**	**Supporting quotations**
Risks and Benefits	“…what percent would get side effects, percent that approved, how rapidly they can expect to see improvement.” *Clinician 4*
	“So I usually give them a highlight of the risk and just the major…the ones that have the highest frequency of occurring and I will go through all of the minor side effects. And then talk about the benefits.” *Clinician 13*
Logistics	“Route of administration is a big deal for patients. So, that certainly is something that really needs to be considered.” *Clinician 3*
	“Usually, I will kind of explain that these are the options and… what potential cost might be.” *Clinician 14*
	“So I tell parents …. [the patient] could be on this medicine for a long time.” *Clinician 4*
Family Preferences	“But it’s their choice, not mine.” *Clinician 4*
	“You’re finding out and trying to understand what would work best on them from a treatment standpoint as far as administering the medication, what their insurance may or may not pay for, what their tolerance or intolerance to the potential specific side effects may be.” *Clinician 3*

When choosing options to present to families, most clinicians always or almost always considered logistical aspects of the treatment such as the route of administration, travel needed for infusions, costs, associated lab tests and the expected length of treatment. However, the specific logistics considered in each situation varied. For example, some clinicians, reflecting on their preferred options, felt that because some treatments only come in one form there is not always a choice to be made about route of administration. Finally, clinicians considered patient characteristics such as age, disease severity and the patient’s psychological and sociocultural context. For example, one clinician identified “chaotic households where there may be two parents or an absent parent, parents incarcerated or…whatever reason” as a particular challenge in treatment decision making.

### Working with families to make decisions

Many clinicians expressed concern about families experiencing information overload. (Table 4) They felt that families’ ability to absorb and retain information is limited, especially at the time of diagnosis. As one clinician said, “…they just are in over their heads and they’re feeling very overwhelmed and you give them a lot of options I think they get—they just don’t know what to do.” Some try to meet the anticipated information needs by providing both verbal and written information.

**Table 4:** Working with families to make a decision

**Sub-themes**	**Supporting quotations**
Information Delivery	“Some people come and as soon as they hear a diagnosis, they can’t comprehend anything beyond that for that visit and then some, the more information you give them the less anxious they are in those kinds of things.” *Clinician 8*
	“I give them data. I show them sheets that have summarized those risks.” *Clinician 9*
	“…some families deal better when you speak in more concrete terms versus other families that, you know, may be more well educated.” *Clinician 2*
Parents’ Fears	“I find it’s harder to implement the injection therapies when the parents are afraid of giving the shot than when the child is afraid of getting the shot.” *Clinician 11*
	“And then also, when you talk about immunosuppressant medications and maybe they’re worried about well what’s going to have an effect the rest of their lives.” *Clinician 2*
	“They might have apprehensions or things like that regarding certain or some of the drugs that we use so that can be inherent or an issue with us getting the medications that we would prefer a patient to be on…” *Clinician 3*
Giving recommendations	“Patients aren’t always amendable to what we recommended.” *Clinician 3*
	“In terms of the type of biologics, I generally recommend etanercept and that is just because we’ve used it a long time, there is data out there, longer sort of term data.” *Clinician 13*
	“And a lot of times, families say, you know, will ask what would you do if it were your child. So, I always say it’s very hard for me to put myself in their shoes but I tell them what I think I would choose if they ask that.” *Clinician 11*

Many clinicians also had concerns that parents’ fears of adverse drug reactions may limit “their willingness to use, maybe, certain medications that I think, perhaps, would be beneficial.” Others felt that addressing parents’ fears led to families being more open to treatment and may provide an opportunity to give the family needed hope and optimism. The concerns about families’ understanding of the disease and treatments, fear of adverse drug reactions and need for hope occurred against a backdrop in which some clinicians worried that families do not or cannot tell them what they do not understand.

Overall, descriptions of the decision-making process largely involved the clinician making a recommendation and anticipating that the family would accept it. However, there were situations that led to clinicians altering their practice, such as families who came to an appointment with outside information, difficult social situations or families with particular concerns about treatment. Likewise, when the evidence was less clear, such as in deciding when to stop treatment, clinicians were more likely to actively consider family members’ preferences.

### Challenges in JIA treatment decisions

Clinicians highlighted a number of medical and system challenges to making treatment decisions for children with JIA. (Table 5) Medical challenges included limited long-term data for some treatments, the challenge of achieving disease control while minimizing adverse drug reactions and long-term risks, and “that we don’t know when it’s okay to stop these medications.” This last point was particularly emphasized during the learning session discussion in which clinicians’ answers about when they would consider stopping treatment for a patient in remission on medication varied widely. There were also health care system challenges; the most commonly mentioned being the cost of treatment and difficulties obtaining insurance approval. Another system challenge was ensuring families get consistent information from all clinicians.

**Table 5:** Challenges in JIA treatment decisions

**Sub-themes**	**Supporting quotations**
Limited long-term data	“They want to know long-term and as these biologics are coming up quicker and quicker, we don’t have some of that data yet and so I think that’s challenging for me.” *Clinician 4*
	“…the unknowns about long-term use of these biologics … I think that factors in too, as far as any decision making that you have. That’s probably the biggest challenge.” *Clinician 14*
When to stop treatment	“You’re really worried about whether when you taper, whether there’s going to be a worsening disease, whether you’ll be able to use the same medications and whether it will be just as effective.” *Clinician 13*
	“One of the questions they always have also is when we stop this medicine which I think is harder to answer because I think we’re better in medicine, at starting medicines than stopping…” *Clinician 11*
Health System Challenges	“I think expenses are a big [challenge], how costly the medication is.” *Clinician 9*
	“…and trying to understand what would work best on them from a treatment standpoint as far as administering the medication, what their insurance may or may not pay for…” *Clinician 3*

## Discussion

Consistent with what is known from parents’ perspectives [6], our needs assessment revealed a clinician-centered decision-making process in JIA, including which options are being considered and the weight given to treatment attributes. Importantly, however, nearly all clinicians described situations in which they would alter their typical process. This suggests a capacity for flexibility and a willingness to consider other approaches to treatment decision making. Such flexibility is essential to PR-COIN’s work to improve shared decision making.

Clinicians’ stated concerns about over-whelming families with information may be one of the reasons that other studies, in both JIA [6] and other conditions [22], have found that families lack desired information. This lack of desired information is particularly notable since the treatment attributes considered by parents [6] mirror those highlighted by the clinicians in this study. Alternatively, due to the different value clinicians and families place on treatment attributes, the relative amount of information provided about each attribute may mismatch the family’s needs. This contrast between clinicians’ concerns about information overload and many families experience of being under-informed [6] suggests that new methods, such as decision aids, are needed for assessing and addressing families’ information needs in pediatric rheumatology.

In adult medicine, numerous studies have addressed patients’ information needs through the use of decision aids [23,24]. In addition to addressing information needs, decision aids may also help ensure that families are included, to the extent they desire, in treatment decisions [23]. Interventions that help develop a two-way flow of information may be more successful at shifting the decision-making structure to one that is more family-centered [25,26], by helping clinicians consider the patient/family goals and preferences, as well as provide sufficient, consistent information to families. Changing clinicians’ behavior will be challenging [27,28], However, our needs assessment revealed opportunities that exist for expanding shared decision making into pediatric rheumatology. For areas that currently lack treatment guidelines or where there is professional uncertainty, such as in treatment de-intensification, clinicians already describe a more active role for families. Developing tools [23,24] that channel this impulse to include families in some decisions is an essential step towards more broad adaptation of shared decision making.

To further facilitate such a change, treatment guidelines could highlight decisions that have multiple medically reasonable options about which patients and parents may have differing preferences. Including such prompts for shared decision making may help alleviate some of the tension between following guidelines and engaging in shared decision making. Additionally, such guidelines could be coupled with decision aids to further facilitate elicitation of family preferences [29].

Given that little research has focused on pediatric clinicians’ roles in treatment decisions, especially among sub-specialists, this needs assessment provides important insight into how such decisions are made but does have limitations. Our finding that information saturation was achieved after the tenth interview was confirmed by the learning session discussion which revealed no new topics or treatment attributes. Although we included clinicians from 12 institutions in our process of data validation the generalizability of this work, especially outside of pediatric rheumatology, may be limited. All clinicians were aware that this work was conducted as preparation for implementing shared decision making tools within PR-COIN. Such knowledge may have influenced the way clinicians discussed decision making, particularly their experiences partnering with families. Additionally, although previous work has focused on parents’ [6] and adolescents’ [10] perspectives on decision making, the study reported here only includes the clinician perspective.

Based on the findings of this needs assessment, the authors of this manuscript and collaborators from PR-COIN are developing a tool to be used during clinic visits to help structure decision making. This tool, like others designed to support selecting a medication from among multiple medications with multiple attributes [24,30,31], will facilitate consistent information delivery and spark conversations between clinicians and families. It will highlight the treatment attributes which providers and parents considered key to treatment decisions. Moreover, the tool will present the minimum amount of information necessary so that clinicians can tailor the depth of information to individual families’ needs. Importantly, while this study presents a needs assessment based on interviews with prescribing clinicians, patients and families will be key partners in assuring our success in developing a tool to support shared decision making.

## Conclusions

Understanding the current decision making process, in this case in JIA, is an essential step towards developing methods for improving the quality of medical decisions. Despite strong evidence of efficacy in adult medicine [23], the adoption of tools designed to foster shared decisions has been slow in practice settings [32-34]. However, by conducting this needs assessment within the confines of a collaborative learning network and garnering agreement about the potential for benefit of such tools, we anticipate a more rapid adoption of tools in network sites and an opportunity to measure their impact. In turn, the adoption of shared decision making tools may lead to improved decision quality and higher value care as chosen treatments are consistent with patients’ and parents’ well-informed preferences.

## Abbreviations

JIA: Juvenile idiopathic arthritis; PR-COIN: Pediatric rheumatology care and outcomes improvement network.

## Competing interests

The authors declare they have no competing interests.

## Authors’ contributions

EL participated in design of the study, conducted interviews and facilitated learning session discussions, coded the data, interpreted the results and drafted the manuscript. WB participated in design of the study, facilitated learning session discussions, coded the data and helped draft the manuscript. JS conducted interviews and coded the data. CL participated in design of the study. EMD participated in design of the study, facilitated learning session discussions, coded the data and helped draft the manuscript. All authors provided critical revision of the manuscript, read and approved the final manuscript. All authors read and approved the final manuscript.

## Supplementary Material

Additional file 1 Interview Guide. Click here for file

Additional file 2 Multiple Choice Questions Used for Data Validation. Click here for file
