# Supplementary material for: Understanding treatment decision making in juvenile idiopathic arthritis: a qualitative assessment
Source: Pediatr Rheumatol Online J. 2013 Sep 30;11:34. doi: 10.1186/1546-0096-11-34 (PMC3849714; doi:10.1186/1546-0096-11-34)
Supplement: Additional file 1 — Interview Guide. [file 1546-0096-11-34-S1.doc]

**Additional file 1: Interview Guide**

*Instructions to interviewer: You may skip questions if they’ve already been answered. Most bullet points contain both a stem question and prompts to encourage elaboration.*

**Section A: Introduction**

To start us off, I’d like to know a little bit about your clinical experience and approach to working with families of patients with JIA.

- What is your specific role in the rheumatology clinic?
- How long have you been in your current position? Where were you before?
- When working with families, what do you think they need to know about JIA? About treatments?
- What do you think you need to know about the patient and his/her family?

**Section B: Decision Process**

As you know, we are interested in understanding how treatment plans are made for patients with JIA. For today, I’m going to focus on treatment care plans for patients with JIA, although if you have examples from working with patients with other conditions feel free to share them.

- To begin with, please tell me your general approach to developing treatment plans in JIA.
- In what situations you find developing JIA treatment plans most challenging. Why?
- Think about a specific situation in which a family struggled with making or implementing a treatment plan. What happened? Is this situation typical of the situations that families find challenging? Why?
- In this situation what did you see as the options that might be included in a care plan?
- When developing care plans, how do conversations between yourself and families typically play out? Are there any particularly sticky or difficult parts of the conversation? How do you handle these parts?
  - Can you give an example of an instance when this may have happened and how you handled it?
- What specific information are you trying to convey to families?
  - How do you, or other members of your team, convey this information?
  - Can you provide an example of a situation where you were recently communicating information to a family and how you went about it?
  - Are there written materials or other resources you provide to families?
  - What feedback have families given you about these materials?
- Is there specific information that you try to elicit from parents or patients?
  - Can you provide an example of how you elicit that type of information?
- What aspects of care plans do families have particularly strong opinions about?
  - Tell me about the last time a family had strong opinions about a care plan. How did you handle that situation? Did you change how you communicated with that family to adapt to their opinions?
- How do you know when a treatment plan has been finalized?

**Section C: Variations in Decision Process**

Thinking about the information you’ve just shared with me. I’d like to go through several specific situations and have you tell me how developing a care plan in that situation is different and the challenges that are specific to that situation.

- What is involved in developing a care plan for a child with newly diagnosed JIA? What is challenging about this situation? Tell me about the last time you developed a care plan for a newly diagnosed JIA. What did you cover? Please explain the conversation you had with that family
- What is involved in revising a care plan for a child with poorly controlled JIA? What is challenging about this situation? Tell me about the last time you revised a care plan for a child with poorly controlled JIA. What did you cover? Please explain the conversation you had with that family.
- What rules of thumb do you use when deciding whether it is time to taper medications? What is involved in revising a care plan for a child experiencing significant side effects on the current treatment? What is challenging about this situation?
  - Tell me about the last time you considered tapering medications for a child with JIA. What was that conversation like? What information did you share with the family? What concerns did your or the family have? Was this situation typical?
- Are any of the situations we just discussed more challenging than others? If so, of all the situations we’ve discussed, which are most challenging for you?

**Section D:** **Decision Outcomes**

- What surprises you either during or after developing a treatment plan?
- What do you think surprises the parents or patient?
- What types of questions come up after the visit? What struggles do families have in implementing treatment plans?
- Tell me about phone calls you, or other team members, have received, related to care plans.

**Section D: Snowball Recruitment**

As you know we are trying to get diverse perspectives in order to create materials that are as useful as possible.

- Is there anyone within pediatric rheumatology that you think we should particularly seek to interview? Anyone who you feel has a different perspective or approach than you do?

**Section E: Wrap-up**

*Interviewer instructions: Have participant complete demographic forms.*

- Thank you very much for participating. Is there anything else you’d like to share with us related to developing treatment plans? Anything else you anticipated we would ask about?
