# Supplementary material for: Understanding treatment decision making in juvenile idiopathic arthritis: a qualitative assessment
Source: Pediatr Rheumatol Online J. 2013 Sep 30;11:34. doi: 10.1186/1546-0096-11-34 (PMC3849714; doi:10.1186/1546-0096-11-34)
Supplement: Additional file 2 — Multiple Choice Questions Used for Data Validation. [file 1546-0096-11-34-S2.doc]

**Additional file 2: Multiple Choice Questions Used for Data Validation**

*Questions were designed for use with Audience Response System*

*Discussion followed* each question to understand the reasons there was or was not variation in clinician’s responses.

| 1. How often do you present route of medication administration (e.g. oral, injection, infusion, etc.) as a factor to consider when starting or changing medications? | Never, Rarely, Sometimes, Almost Always, Always |
| --- | --- |
| 1. How often do you present the frequency of medication administration as a factor to consider when starting or changing medications? | Never, Rarely, Sometimes, Almost Always, Always |
| 1. How often do you discuss out-of-pocket costs when starting or changing medications? | Never, Rarely, Sometimes, Almost Always, Always |
| 1. How often do you discuss potential side effects when starting or changing medications? | Never, Rarely, Sometimes, Almost Always, Always |
| 1. How often do you discuss the likelihood of the medicine working when starting or changing medications? | Never, Rarely, Sometimes, Almost Always, Always |
| 1. In general, how long do you tell parents it will take before a medication can be judged a success or a failure? | < 3 months, 3-6 months, 6-9 months, 9-12 months, > 12 months |
| 1. In your clinic, which approach is typically used to education families about injecting medication? (choose all that apply) | Verbal presentation, Printed materials, Hands-on demonstration, Video demonstration, Peer to peer education, Other |
| 1. How often do you change medications because a patient or child objects to current route of administration? | Never, Rarely, Sometimes, Almost Always, Always |
| 1. In general, how long does a patient need to be in remission on medication before you suggest stopping or tapering medication? | < 3 months, 3-6 months, 6-9 months, 9-12 months, > 12 months |

| 1. How often is your decision about the timing of tapering medication influenced by the patient’s original disease severity? | Never, Rarely, Sometimes, Almost Always, Always |
| --- | --- |
| 1. Scenario: Your patient has recently become symptom free and has no arthritis on exam. The parents want to stop treatment as soon as possible. How do you respond? | Now, 3 months, 3-6 months, 6-12 months, 12-18 months, >18 months |
| 1. How accurately does the following statement describe your prescribing approach: “For the majority of patients, I have a preferred approach to treatment (eg. early aggressive therapy, step-up therapy, follow published guidelines)”? | Strongly disagree, Disagree, Neutral, Agree, Strongly agree |
